# Hints for a Medical Topography of Great Britain

**Published:** 1809-02

**Authors:** W. Royston

**Affiliations:** Prince's Street, Cavendish Square


### HINTS FOR A MEDICAL TOPOGRAPHY OF GREAT BRITAIN.

### *In a Letter addressed to Dr. ADAMS.*

( Continued from our last. )

WE have seen a most destructive fever of the present time referred to an atmospherical influence, or to an air impregnated with some poisonous material; but the qualities of that material have never been detected, and its origin has only been guessed at. Perhaps, on the spot where the air was supposed to be imbibing or absorbing this generator of fever, it has not been chemically examined. A high temperature of the atmosphere is, however, universally admitted to be a *sine qua non* of the existence of this fever; when the thermometer sinks to a certain point it is no longer seen. Yet it must not be concluded that heat alone is the pabulum of this disease; but rather that a high temperature is essential to the evolution of some obscure and hitherto unknown poison.*

Are there local peculiarities that produce diseases proper to certain places? In the low parts of England called *Fens*, the frequency of intermittents is very remarkable. The counties of Cambridge, Essex, Huntingdon, and Lin-

H 2 coln,

* In examining the state of the air of any particular place, with a view to ascertain its peculiar qualities, it seems essential to attend to two very distinct points. The variations in the quantities of its constituent parts, and the adventitious admixture of certain supposed deleterious effluvia. Every 100 parts of sufficiently pure and respirable atmospheric air is composed of 22 parts of oxygen, 77 of nitrogen, and 1 of carbonic acid gas. It will be easily understood that many deviations from these proportions may constitute an atmosphere unfavourable to animal life; and that these deviations may be rendered more destructive by being combined with animal effluvia, marsh miasmata, and the seeds of epidemics. But the utmost exertion of medical philosophy has not yet explained this difficult and obscure subject.

coln, abound with a kind of marshy land having some specific property, or furnishing some active principle that gives rise to this genus of fever. But what this principle is, has not yet been discovered. It has been distinguished by the term marsh *miasma*; and it is certainly something more than mere humidity, for it is not generated in all low-lands. There are districts on the banks of our principal rivers frequently inundated, and of course very humid, where an intermittent is seldom seen. Of this the Thames affords some instances. The progress of this disease has also been observed to undergo a suspension, when the floods that at times descend rapidly from the up-lands, spread an immense sheet of water over these fenny districts. The season for the appearance of this fever, is not that most remarkable for humidity. After the unusual heat of the last summer, the frequency of intermittents in the autumn was increased in the fens of Cambridgshire to an almost unprecedented degree; and even quadrupeds were not exempt, for distinctly marked cases of *tertian* were observed in horses. In the year 1780, a similar prevalence of this disease occurred in the same part; and though in an interval of 28 years many and frequent sporadic cases had arisen, yet its universality, during that period, was suspended. We have to regret, Sir, that a correct record of the constitution of the year 1780, as applying to this particular district, has not been preserved in such a manner as to admit of a direct comparison with that of 1808. If it were possible, from authentic documents, to compare the history of these two seasons, much light might be thrown on the obscure cause of intermittents. As it is, if the defect is properly felt, it will impress the Faculty with a due sense of the demand that Society has on them, to record every fact connected with the extraordinary prevalence of disease; whether directly as it regards the morbid appearances themselves, or indirectly as it has a relation to their cause, involving the general circumstances of natural history, the state of the atmosphere, the growth of vegetation, and incidents connected with many temporary and local particulars.

The causes that produce intermittents may possibly influence other parts of nature, and produce effects unusual in kind or degree. In a short visit to the Fens of Cambridgeshire in last November, it was observed that the hedges of the *Cratægus oxyacantha* were universally covered with the web of the caterpillar of the *Papilio cratægi*, to a degree of which there is no previous record; and that that the winter fruits had an extraordinary disposition to decay. The *rot* in sheep often prevails to an alarming degree, in the up-lands that skirt these fens. It is however a curious fact, certainly not known to a late investigator of this subject, who refers intermittents and this disease of sheep to the same cause, that the *rot* never occurs,* at least is never generated, on the lands properly denominated *Fen*.† The

* I do not mean to assert this fact extends beyond the Fens of Cambridgeshire and Huntingdonshire: it probably, however, applies to all fenny lands. But this your numerous correspondents have ample means of ascertaining. It is interesting to science and to society to know whether *Rot* in sheep is ever generated on Fen-lands; and if it is the practice of farmers to run their sheep on such lands, with a view to suspend or cure the disease, if not in a very advanced stage. It is precisely that character of land, lying by the sides of rivers or subject to be frequently overflowed by up-land brooks, and where intermittents do not commonly occur, that produces the *Rot* in sheep.

† The district here meant, as properly denominated Fen, is that immense level of 300,000 acres, which stretches away from the high-lands of Norfolk, Cambridge, Huntingdon, and Lincoln, to the coast of the latter county at Boston. In this extensive flat there must be varieties of soil: there are two, however, which prevail, distinguished by the provincial terms *Moor* and *Silt*. The *Moor* soil prevails on the land side. In colour it approaches to black, is extremely loose in its texture where it lies on the surface, scarcely admits of cultivation, but is cut into a kind of fuel called *Turf*. It is considered to have been originally a deposition from water, and is now endowed with a power of increase analagous to vegetation. It lies from 2 to 5 feet thick on a stratum of clay, once probably meadow; and is covered with various thickness of extremely fertile vegetable mould. In this district intermittents are always sporadic, and at times, increase by the operation of occasional causes, to an universal prevalence; but the *Rot* in sheep is never generated.

That part of this district which lies toward the sea, presents a specimen of soil extremely different from the former. In colour and texture it appears a fine brown sand, susceptible of an extraordinary degree of fertilization, producing corn and grass in great abundance, and grasses especially, of the finest quality. This soil, under the denomination of *Silt*, has, probably, been formed by the flux and reflux of the sea, which at every flow of the tide would deposit some portion of this substance. At a very remote period, perhaps while the Romans possessed Britain, the flow of the sea over this part of the Fen was prevented by an artificial rampart; and an apparently sandy waste was converted by degrees, and under the operation of a variety of causes, into the most fertile and productive portion of the British Islands.

The Naturalist, the Botanist, and the Physician, will find their researches in this district not misapplied. A great variety of water fowl, both waders and swimmers, are here met with. The *Ardea major* and *stellaris* are common; and the singular spring note of the latter gives an interest to the scenery of these extended flats.

"*The Bittern* booms along the sounding marsh."

The *Scolopax gallinago*, *gallinula*, and *ægocephala*; *Tringa canutus*,

 *pugnax*,

The production of fever by peculiar states of the atmosphere, or by its conveying the pabulum of that disease to the lungs, though a circumstance deserving great attention, is not the only one in which an atmospherical influence operates to the production of disorder in the actions of animal life. In no other state of the system is this influence more decidedly marked than in those morbid actions of the lungs constituting asthma. Some asthmatics bear the air of London best, others that of the Country; some are free from the paroxysm during the cold of winter, others in the heat of summer. Temperature, humidity, chemical impregnations, or deviations from the standard in the constituent materials of the atmosphere, may separately or conjunctly operate on these invalids. They are always morbidly sensible to every kind of effluvia; and in some instances this sensibility has an inconceivable acuteness. A man of large make, great muscular strength, conjoined with mental inactivity, and subject to asthma, was so irritable to the effluvia rising from ipecacuanha, that he

---

*pugnax*, and *erythropus*, several species of the genus *Fulica*, as the moor hen and coot: the *Sterna hirunda*, many species of *Larus*, and a great variety of the genus *Anas* are very common. The pools of almost stagnant water, some miles in circumference, called *Meers*, abound with various species of fish, some of which grow to a great size, as eels and pike. Those genera of flies which pass a considerable portion of their existence in stagnant water, are found here in great abundance. The botanist will have his labours rewarded by the discovery of numerous tribes of aquatic plants, many of them curious from their structure, habits, properties, or form. That beautiful little plant the *Parnassia palustris, the Nymphæa alba, Butomus umbellatus, Eriphorum polystachion*, and *vaginatum* are frequent. The physician must be interested by the opportunity of studying the cause, symptoms, and cure of intermittents, which their frequent recurrence here affords. There are also some remarkable, and, perhaps, yet unnoted diseases among cattle, believed to arise from feeding on the natural herbage of these marshes. The progress of agriculture is, however, so rapidly approximating this region to the features of the surrounding country, and its native character is so fast wearing away, that a very short time will obliterate its distinguishing marks; and the next generation will search in vain for facts that are now so valuable to the naturalist, as the present looks back to the period, when this district was described as an inpenetrable morass, covered with an enormous growth of the *Arundo phragmites, Calamagrostes, Epilobiums*, and *Carexes;* affording only a retreat to innumerable flights of water fowl, and having its *Meers* stocked with immense shoals of fish. In the few spots to which the persevering industry of man had then penetrated, he was himself pictured as uncultured and rude, almost amphibious, going upon stilts, and partaking of the wild character of nature that surrounded him.

he was unable to remain in a house where the pulverization of that vegetable was performing.

That there are some places of great and nearly permanent salubrity in England, as there are others unwholesome, and very constantly visited at some seasons of the year with endemial diseases, will not be denied. But whether the salubrious or unhealthy peculiarity depends on some direct quality in the atmosphere, arising from a want of proper balance among its constituent gases; or indirectly, from having mixed with it some unknown exhalation, in a gaseous or other form, is still a *desideratum.* Can this point be determined in any other way than by actual experiment on the air of different places? Some part of Derbyshire is unquestionably salubrious in a degree unknown to other districts; and, perhaps, so absolutely healthy as to afford a standard of comparison for all other places. But before this standard can be practically applied, it is essential to know of what it is constituted. It seems not difficult, at least in many important points, to ascertain this with sufficient precision. The elevation, qualities of the water, state of agriculture, and many circumstances before mentioned, may readily be determined. A practical chemist will have no difficulty in shewing the qualities of its atmosphere, and possibly detecting many incidental impregnations, or demonstrating its freedom from such contamination. The habits, customs, and diet of the inhabitants are obvious to explanation. When these, and perhaps many other facts which will arise to an active inquirer, are ascertained with scientific precision, we shall possess a determinate point, a standard with which to compare the examinations of other districts less healthy; tracing every deviation from this standard, and its corresponding diseases, until the actual state of every part of the kingdom, as to salubrity or the contrary, is decidedly known.

In making this statistical inquiry many extraordinary cases of disease, or singular idiosyncracies will be discovered. Though it may not generally be so advantageous to science to record these isolated facts, as to develope the cause and course of disease, and the more humble but common operations of nature; yet such singular occurrences should not be neglected, for they may unexpectedly explain some principle, or unfold some obscure trains of action. If to the faithful record of usual appearances science is indebted for her most rational principles, events out of the common course of nature may, at times, elucidate uncertain facts, and afford an exposition of abstruse laws. But the fascination with which these extraordinary cases act, especially upon the younger parts of the profession, should be resisted with a fortitude that a just sense of the utility that arises from recording ordinary occurrences only can supply: otherwise, uncommon facts may, by abstracting the attention from the plainer course of observation, retard the progress of medical discovery.

Among these rare cases, none have excited the interest of mankind so much as *Longevity*. To procrastinate the period of old age, and of final dissolution, the reasonings of the dogmatist and the experience of the empiric have been called forth. For this the Galenist endeavoured to reconcile the jarring elements, and poise the contending principles of hot and cold, dry and moist: for this the alchemist tortured every material of nature, until he fancied that his "operations by fire" had given him the precious elixir that was to restrain the hand of fate. But experience has explained that neither the theses of the Schools nor the laboratory of the chemist, have yet afforded the means of parrying the inevitable blow. If art is ever to teach the modes of prolonging life, she must look for her principles in the practices of the *Cornaro's* that have at distant periods, and in sparing numbers, surprised the world. On this principle, whenever instances of longevity occur, it will be essential to ascertain if they are hereditary, and connected with any peculiarity of structure; induced by habits of life; bestowed on the mountaineer, or given to the inhabitants of vallies: whether in a dry or a humid region they are most frequent.

If the desire for length of days has occupied the feelings of mankind in an uncommon manner, a few instances of living without food has excited proportionate surprise and curiosity. There are on record several extraordinary instances of abstinence; and in your Journal for last November, a case of this kind has deservedly attracted considerable attention. But strange as this power of existing without sustenance may appear, it is, perhaps, less rare than has been suspected. In a remote part of North Wales there is actually at this time a counterpart to the case reported by Mr. Taylor. This extraordinary being is also a woman, named *Mary Thomas*. She is now of the age of 84 years; 63 of these she has been confined to the bed; and during this long period has lived nearly without eating or drinking. For ten years, about the middle of this long term, she was supported absolutely without food of any any kind; then lying in a torpid state, unconscious of her own existence. In 1807 her *ingestæ* were confined to one ounce of bread and a glass of water in fourteen days; and this was invariably rejected from her stomach in a few minutes after being taken. Under this extended period of abstinence she is reduced to a breathing skeleton. An eminent artist, Mr. James Ward of Newman Street, has in his possession an admirable sketch, unique in its kind, of this being, taken from the life by himself. It is superfluous to observe how much philosophers and physicians would be gratified by an etching from this curious portrait, executed with the truth and spirit its possessor is capable of giving to it; and accompanied with such authentic facts, as Mr. Ward's knowledge of the woman can supply. I will venture to assert that such a document would find a ready admission into the Medical and Physical Journal.

From these aberrations, the medical philosopher, gratified even by extravagancies that manifest the infinity of powers and endless varieties of nature, will descend with increased zeal to subjects of more obvious utility, as well as of more frequent occurrence.

The prevalence of *Hernia*, especially in that class of society who live by labour, will determine every benevolent inquirer to make exertions to ascertain the cause of this disease; and thus afford the means of prevention to a certain extent. It will be a question if the disease at all depends on locality, if connected with low, damp, and what are supposed relaxing situations; if it is at any time hereditary, and then depending on original organic defect; occasioned by any particular employments, induced by intemperance, or in any degree influenced by methods of nursing children among the lower classes. The frequency of *struma*, and that unmanagable form of it *phthisis pulmonalis*, will impel to investigations that may determine in what districts it is most rare; or whether the sea-coasts, especially of the western parts of the island, afford a probable security against the attack of consumption in those habits manifesting that organization which constitutes hereditary predisposition. *Cynanche trachealis* seems to be governed by certain peculiarities of situation. In some places it is almost an endemical disease, in others it hardly occurs. A practitioner of sufficient intelligence, met with but two cases in twenty years extensive practice in the fenny parts of Cambridgeshire. If this disease is purely inflammatory, its occurrence may be regulated by those localities calities that influence this diathesis; and thus marshy places may be unfavourable to its production. But *pneumonia* is certainly not uncommon in the district where *cynanche trachealis* did not appear. This fact renders the question of its cause as at all depending on situations still more obscure.

To particularize the multitude of effects on the health that popular opinion attributes to some state of the atmosphere, or to some local properties, many of which have the assent, at least, of medical practitioners, would occupy an extensive volume. Enough, perhaps, has been said to induce the Faculty to inquire into the existence of these effects, and their cause; as well as the *modus operandi* of that cause.

The diseases occasioned by various mechanical and agricultural employments, by chemical operations, &c. are less latent and obscure than those believed to arise from particular states of the atmosphere. The investigations of Ramazini, Falconer, Kirkland, Skragge, &c. have still left much to be done on this subject: and practitioners so stationed as to have the diseases thus produced frequently under their observation, will essentially benefit the science of medicine, by recording from time to time, their symptoms, modes of treatment, and termination; as well as their cause. It is presumed that Sheffield, Birmingham, the mining counties, manufactories of pottery, the salt works of Cheshire, &c. will present many of these peculiar morbid derangements.

The *Veterinary Art* has assumed a form, in this country, that entitles it to a place among the liberal studies. Diseases that were once under the managment of the *cow-leach* and the *sow-gilder*, are now treated by men regularly educated, and among whom are found many of extraordinary acquirements both in natural history and science. We cannot, Sir, but look to persons thus accomplished, and who are scattered over various parts of the island, for interesting information in their own department. There are yet diseases among cattle not sufficiently understood. The ravages occasioned by the *rot* in sheep has not hitherto been checked by any knowledge we have of that disease. It has been observed in the preceding pages, that there are distempers among cattle in the fenny districts of this country, very little known; and the history and description of these morbid appearances will be most correctly given by those who reside on the spot, and who have frequent opportunities of observing their progress. The gentleman who amuses himself with agriculture, the intelligent farmer, mer, as well as those who profess the Veterinary Art, may furnish information on these subjects of infinite consequence.

A general view of the state of medical practice, will form an important addition to the local descriptions, natural history, and diseases.

The multitude of practitioners scattered over this country, are comprehended in two classes:—*regulars* and *irregulars*. An inquiry into the actual circumstances of each of these classes, will probably bring out many curious and valuable facts. It is evident, however, that at present an acquaintance with them is very imperfect. Several efforts have been made to reform and meliorate the practice of medicine, and improve the condition of its professors; all of which have failed, possibly from the want of a distinct knowledge of its existing state. It will not be denied that whenever a judicious reform takes place, it is founded on a thorough previous acquaintance with the real circumstances of the subject of reformation. This essential degree of knowledge cannot be collected, however, on a multitude of local particulars connected with medical practice, but by the joint efforts of those whose stations must make them fully informed on those particulars.

With the first class, or *regular practitioners*, many circumstances of great interest are directly connected. The numbers engaged in actual practice, as physicians, surgeons, apothecaries, or accoucheurs; the separate or consolidated exercise of these different departments; the proportion of remuneration allotted to each:—the institution, history, and management of hospitals and dispensaries;—the conduct of county and parochial poor houses in their medical department;—public and private receptacles for lunatics, are subjects that cannot fail to be noticed in a general survey. Of no less interest will be accounts of the modes of conducting and exercising the different branches of the profession; the history, views, and regulations of medical and philosophical societies;—public and private medical libraries, with descriptions of scarce or valuable printed books and MS.; societies formed for the benefit of decayed members of the medical profession, their widows and children; when and by whom instituted, their present condition, and the laws by which they are governed.

There is a branch of knowledge intimately connected with the respectability of the medical profession, which has been so unaccountably neglected in this country, that when it was lately an object of discussion in the house of commons, commons, one of the most reputable members of that assembly professed to believe that not only the subject itself was inconsequential, but that its name was absurd and contradictory. But few, perhaps, Sir, will admit that the code of principles which is intended to guide the practitioners of medicine in their decisions upon cases of the highest importance in civil society, is either insignificant in itself or absurdly distinguished by the term *Medical Jurisprudence.* Let this code, however, be designated by any other name, if any other can be found more appropriate; if the code itself be sufficiently full and distinct, to enable practitioners, when called into courts of justice, to give evidence with the precision and certainty that is expected from the members of a learned profession. The dearest interests of society and of individuals are frequently implicated in the professional evidence of medical persons. Life, reputation, or property, are often involved in these decisions. Of what serious importance is it then to be instructed on this subject: and though we look forward with a well founded hope to the elucidation it is to receive from *Professor Duncan,* still the inquiries of the most learned may be assisted by industrious collectors of facts; and, perhaps, even the idle may be stimulated to exertion by the apprehension that their mistakes may be liable to public animadversion. The objects of medical jurisprudence are various. In criminal, civil, and consistorial courts, questions occur in which the medical practitioner must either be distinguished by his knowledge, or dishonoured by his ignorance. In the first of these courts, the most frequent, difficult, and important questions will arise on cases of *infanticidium, stuprum violentium,* and *abortus procuratus.* The various species of homicide, and the legal inspection of dead bodies, also, are circumstances of great moment. The questions before civil courts, though less awful because they do not affect life, are often more intricate, being in their nature obscure, and generally connected with some mysterious design. *Insania* in all its species; fatuity; pregnancy, simulated or concealed; labour concealed, assumed, accelerated, or retarded; diseases alleged, imputed, or hidden, may all require to be elucidated, and substantiated, by the evidence of the faculty. Impotence in the male, sterility in the female, uncertainty of sex, and syphilitic disorders, as the ground of divorce, are frequently questions of as much difficulty as importance.

The class of irregular practitioners will attract attention, because it is desirable to know to what extent empiricism is carried, carried, to ascertain the character and history of the most celebrated quacks, the ground of their popular fame, and the validity of their various pretences. It is something more than idle curiosity that will endeavour to ascertain whether unusual cures are ever performed by them, and if so, what have been the means employed; whether they have arisen from accident, been effected by strong mental impressions, or performed by some secret medicine of uncommon efficacy. It might probably turn to some account, not only to discover what these secret medicines are, but to explain the usual course of practice of the most noted empirics. It is well known that most extraordinary and extravagant means are used by these persons, with a success as great as unexpected: and it is certainly desirable to give a full explication of those means. It is not designed here to characterize the vendors, or even the inventors of patent medicines, but the daring adventurers that are sometimes seen to spring up in every district, who, by the mere operation of native talent, taking advantage of some fortunate hit, rise into sudden and extensive reputation. But observations on this subject should ever be guided by candour and moderation: they belong to truth, and the fair desire to collect information. Angry invectives against quackery, splenetic remarks, the affectation of wit, and the petulance of criticism, are born of another spirit, and seldom serve the cause of science.

When I look back on the number of pages I have occupied, in a work devoted both to receive and to communicate knowledge, I feel that I have no better apology to make than my desire of being useful; and to lament that my powers are not adequate to my wishes. But if this view of a very interesting subject be thought by some too much extended, others may think it has suffered by contraction: I am myself sensible, that much should be added to make it sufficiently explanatory, of a design comprehending circumstances that benefit society in proportion to the clearness with which they are understood. In its present form it is to be considered, however, as no more than a hasty outline, left to be corrected and filled up by the resident Faculty of Britain; who only are competent to bring the plan to that decisive perfection that may render it permanently useful. Yet on many points, persons not of the medical profession, may furnish valuable matter. Gentlemen who reside much on their estates, men of general science, and especially the *parochial clergy*, may, by the communication of facts in *natural history, local descrip-*

*tions,*

*tions*, the *flora* of districts, extraordinary deviations from the course of *Nature*, the prevalence of *empiricism*, *&c. &c.* materially assist the design: and I cannot, Sir, for a moment doubt but your JOURNAL will promptly afford the medium of giving such intelligence to the PUBLIC.

W. ROYSTON.

*Prince's Street, Cavendish Square,*
*December*, 1808.

